# Morphological Transformation between Flat and Tube Structures by Coordinated Motions of Soft Pneumatic Microactuators

**DOI:** 10.1038/s41598-019-50670-7

**Published:** 2019-10-09

**Authors:** Satoshi Konishi, Fumitaka Oya

**Affiliations:** 1 Ritsumeikan University, College of Science and Engineering, Department of Mechanical Engineering, Kusatsu, 525-8577 Japan; 2 Ritsumeikan University, Graduate School of Science and Engineering, Kusatsu, 525-8577 Japan

**Keywords:** Electrical and electronic engineering, Mechanical engineering

## Abstract

Microactuators are the most distinctive and challenging microdevices among micro electromechanical systems (MEMS) relative to microsensors or electronic circuits. Soft and flexible microactuators have been achieved by introducing polymers as structural materials in addition to conventional materials. Expanding the application of MEMS to the biomedical field requires particular features, such as softness and small devices. It is important to address small and fragile biological objects while satisfying the demand for minimally invasive medicine. Both MEMS and biomedical applications require three-dimensional microstructures for higher-order functions. In general, microactuators are limited to simple motions such as bending. Our group has developed an openable artificial small intestinal tract system. An array of pneumatic balloon actuators (PBAs) transforms a flat structure into a tube structure representing the small intestine. Coordination of the bending motions of the PBAs enables the formation of a three-dimensional tube structure. Each PBA is 400 μm × 1800 μm × 100 μm. The diameter of the tube structure is 1 mm. Additional higher-order functions of the artificial small intestine, such as peristaltic motion, are currently of interest for us. This paper describes the morphological transformation of a soft microstructure and further potential possibilities of coordinated motions of soft microactuators.

## Introduction

This paper describes a morphological transformation of the microstructure by soft pneumatic microactuators. The simple motion of microactuators can be combined and arranged to provide a higher-order function. A flat film integrated with an array of pneumatic balloon actuators (PBAs) is transformed into a tubular shape by the bending motion of PBA arrays. In general, most microactuators provide simple motions in applications of micro electromechanical systems (MEMS). Parallel plates attracting each other through electrostatic forces and a comb drive actuator^1^ can generate linear actuation. A digital micro mirror device (DMD) can rotate around a torsion bar by applying an electrostatic force between the mirror plate and the substrate^2^. A multimorph structure, such as a bimorph structure, bends due to the difference in the strain of layers. Bimorph actuators can bend based on various principles. For instance, thermal and piezoelectric principles are applied to generate the strain and actuation of bimorph structures^3,4^. Piezoelectric bimorph actuators have long been commercialized. A thin film of a shape memory alloy, which is categorized as a thermal mechanism, is also used to implement bending actuation^5^. This work is focused on pneumatic actuation. A PBA applies pneumatic pressure to an inflatable balloon to generate strain^6^. The first type of PBA employed a combination of elastic polydimethylsiloxane (PDMS) and nonelastic polyimide films. Another group reported the combination of parylene-C and Si^7^. This actuator could generate high force through high pressure owing to its high pressure-resistant structure. Next, an all PDMS PBA was developed^8^. The third generation of PBAs can be manufactured by a simple process of molding and bonding. Most interestingly, this PBA is soft enough to actuate by applying relatively low pressure.

The coordinated motions of microactuators have been studied to overcome the classical limitation of the simple motion of microactuators in MEMS. Comb drive actuators stack a large number of elemental teeth electrodes to generate a large electrostatic force. A DMD utilizes a large number of tilting micro mirrors to generate a cooperative work of pixels for the display^2^. The ciliary motion system is one of the most typical approaches for the motion coordination of microactuators^3^. In a ciliary motion system using a bimorph thermal microactuator, two sets of cantilever-type actuators are arranged opposing each other. Two sets of cantilevers move downward by turns at different phases. The coordination of a large number of sets can carry a small piece of silicon wafer. Similar demonstrations have been reported by many groups^9,10^. The first generation of PBAs was also arranged together with a ciliary motion system, and they could carry a silicon chip^6^. A ciliary motion system employing a thermal bimorph actuator was developed as a two-dimensional conveyance system based on the concept of distributed micromotion systems^11^. These works succeeded in rendering complicated or higher-order motion through coordination of the simple motions of microactuators. Microactuators have been applied to microvalves and micropumps in fluidic MEMS. A diaphragm pump uses the simple vibration of a membrane together with a combination of check valves^12^. The diffuser pump was presented as a valve-less pump^13^. A peristaltic micropump uses a phase difference in vibration with a series of diaphragm pumps and generates peristaltic pumping as higher-order motion^14^. A peristaltic pump has the advantage of a simple design in comparison with a micropump using active check valves. Furthermore, we improved the design of a pneumatic peristaltic micropump and reported single-phase pumping^15,16^. Recently, various driving principles of peristaltic pumping, such as electro-magneto-hydrodynamic pumping^17^ and even bioactuated pumping^18^, were newly developed, whereas continuous design improvement of the traditional piezoelectric principle has been studied in terms of dead volume and response time^19^.

In the field of robotics, inflatable actuators are applied to provide motions such as locomotion^20^. A ciliary motion system can walk when the system is placed upside down on the ground. Our group presented twisting motion of microactuators by coordinated motions of PBAs^21^. PBAs were arranged with a certain angle formed by the individual longitudinal directions of the whole structure with respect to the PBAs. Bidirectional twisting motion could be demonstrated by combining PBAs arranged with different angles.

Most of the microstructures in MEMS based on thin film technology are planar in nature. Various efforts have been made to fabricate three-dimensional microstructures beyond the inherent limitation of thin film technology. A hinge mechanism was designed to build three-dimensional microstructures from micromachined planar structures^22–24^. Micromachined hinge structures were assembled using thermokinetic force^23^. Ferromagnetic microstructures with elastic hinges were assembled by an external magnetic field^24^. An origami technique has inspired various assembly processes in the MEMS field. Origami-like folded MEMS were developed for an inertial measurement unit^25^. Self-folding structures were developed to create a three-dimensional encapsulation container for bio particles^26^. Origami biosystems created by bending, curving and folding of molecules or thin films have been reported^27^. Moreover, microactuators have been used to assemble planar microparts fabricated on a substrate to construct three-dimensional MEMS. A scratch drive actuator was used to move and assemble microparts on a substrate^28–31^. Many optical MEMS structures were completed by assembly.

In the field of biotechnology, *in-vitro* cell or tissue engineering requires three-dimensional cellular aggregates for biomimetic modeling. Conventionally, cultured cells are planar in a dish or micro-well. Three-dimensional cellular aggregates that form a spheroid became popular as an *in vitro* biomimetic model. The concept of an organ-on-a-chip explores further advancements for *in vitro* biomimetic modeling^32,33^. A blood vessel was constructed by cultured cells in a fluidic microdevice^34^. A study on gut-on-a-chip reported two layered microchannels with a filter between the upper and lower channels on which the cells were cultured^35^.

Based on the background described above, our group reported an openable artificial small intestine tract system using PBAs^36^. An array of bending PBAs transformed an initial flat shape into a tubular shape. The openable artificial small intestine tract system was developed as an organ-on-a-chip. Drug absorption by Caco-2 cells on its internal wall was evaluated by perfusion flow evaluation through the tubular structure. Cells in the tube could be observed or even sampled by returning the structure to the flat state. Additional higher-order functions of the artificial small intestine, such as mechanical stretch and peristaltic motion, are currently of interest for us. This paper describes the morphological transformation of a soft microstructure and further potential possibilities of coordinated motions of soft microactuators.

## Results

### Conceptual design of a transformable structure having an array of bending PBAs

**Figure 1:**
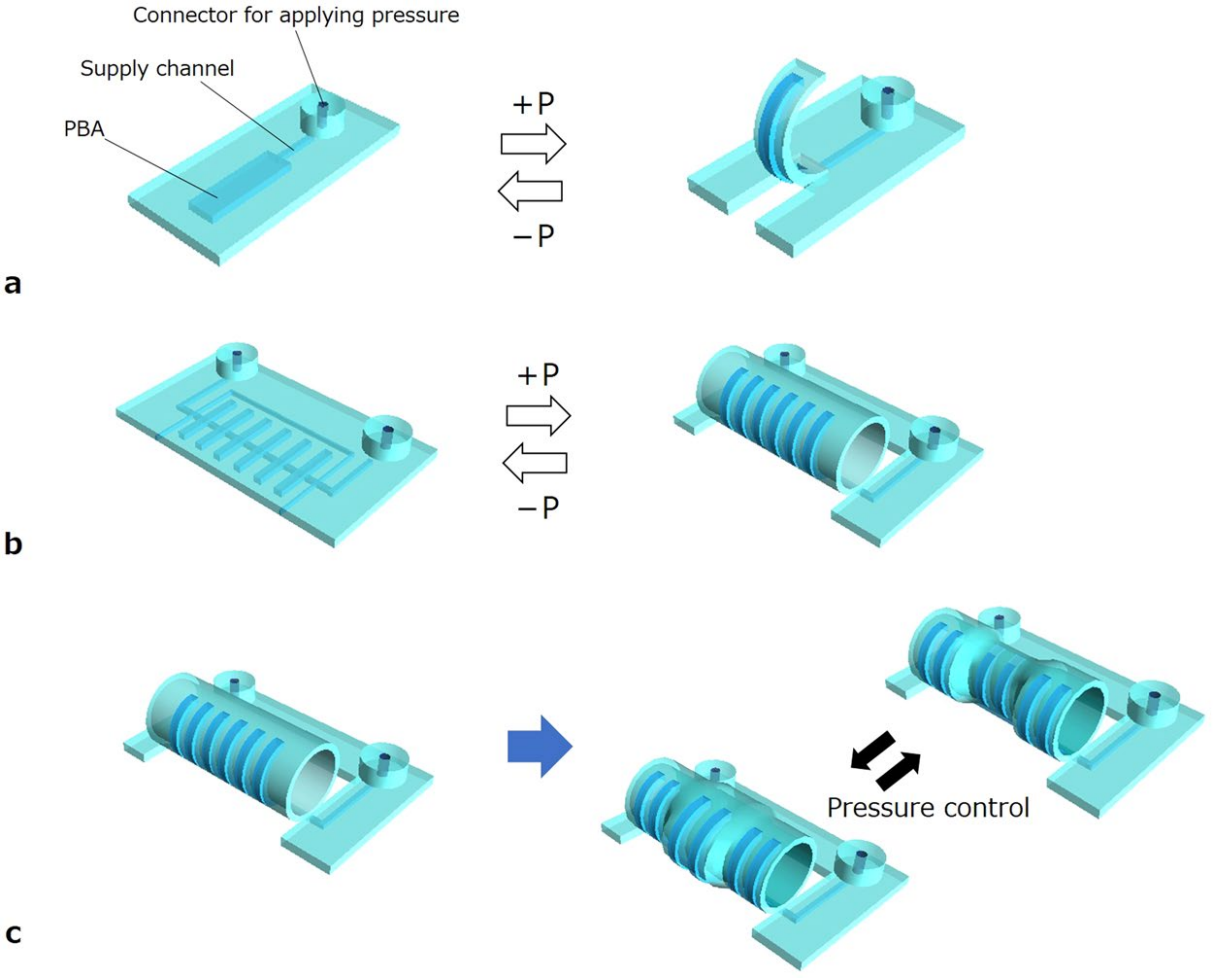
Coordinated motions of soft microactuators for morphological transformation. (**a**) Elemental bending microactuator (PBA). Bending PBAs bend due to the strain difference caused by an increase in internal pressure. (**b**) Transformable structure composed of two opposing films haing an array of PBAs for each. A tubular shape is formed from a flat structure by pressurization. (**c**) Independent motion control of PBAs for higher-order motion such as peristaltic motion of tube structure. The applied pressure to PBAs in the central section of the tube structure is controlled to generate expansion/contraction in the illustration.

Figure 1 presents conceptual illustrations of the coordination of simple motions of microactuators turned into higher-order motions in this work. In particular, this study uses a bending PBA as an elemental actuator (Fig. 1a). Bending PBAs bend due to the strain difference caused by an increase in internal pressure. Its motion principle is similar to that of a bimorph actuator. An openable artificial intestine was designed and applied to drug screening in a pharmaceutical study^36^. The openable artificial intestine exploits the cooperative motions of an array of PBAs to transform the initial flat structure into a tube structure. Figure 1b presents the transformation from the flat to tube structures. PBAs were arranged in parallel and connected together to form a flat film. One of the sides of this film was fixed to the substrate. The PBAs were driven in batch by pressurization so that the film rolled up. A tubular shape was completed by combining two opposing films, as shown in Fig. 1b. The initial flat structure was suitable for deploying cell cultures on it. The tube structure is advantageous in regard to reproducing biomimetic conditions in perfusion evaluation for drug screening. Furthermore, the peristatic motion is attractive from a biomimetic point of view for its application to the artificial small intestine. The independent motion control of PBAs is designed to implement higher-order motions. Figure 1c illustrates the design of independent motion control of individual sections composed of PBAs. This paper reports the design and characterization of morphological transformation between flat and tube structures using soft PBAs. The independent motion control of PBAs integrated into the transformable structure will be described in terms of higher-order motion through coordination of simple motions of microactuators.

### Fabrication results of the transformable structure

**Figure 2:**
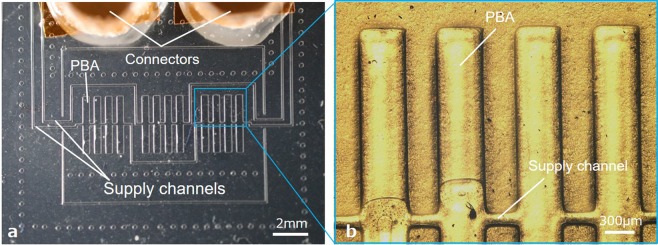
Fabrication results of the transformable structure having an array of bending PBAs. (**a**) Photograph of a top view of an initial flat shape. Two opposing films having twelve PBAs and supply channels are arranged. Two connectors are placed at both ends of the structure to supply pressure to the PBAs. (**b**) A magnified view. Each PBA is 400 μm × 1800 μm × 100 μm. A regular spacing between the PBAs is 300 μm.

A morphologically transformable structure made of PDMS was fabricated on a Si substrate (Fig. 2). An array of PBAs was fabricated in a PDMS film that was fixed at one side on the substrate. Two opposing films were arranged to connect at their fixed side on the substrate. Figure 2a shows a photograph of the top view of an initial flat structure. Each film is 3 mm × 11 mm × 100 μm. In total, twelve PBAs are arranged with a regular spacing of 300 μm in each film. Two connectors are placed at both ends of the structure to supply pressure to the PBAs. A magnified view of an array of PBAs is shown in Fig. 2b. Each PBA is 400 μm × 1800 μm × 100 μm.

### Morphological transformation from flat to tube structure

**Figure 3:**
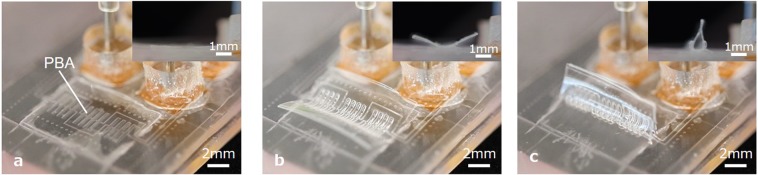
Tube structure transformed from the initial flat shape. A side view is inserted in the top right corner of each photograph. **(a)** The initial flat shape. The applied pressure was 0 kPa. **(b)** The middle state between flat and tube shape. The applied pressure was 30 kPa. (**c)** Tube structure transformed from the flat shape. The applied pressure was 40 kPa.

The PBA bends due to the internal stress when it is pressurized. Figure 3 shows sequential photographs of the morphological transformation from a flat film to a tubular structure. A side view is inserted in the top right corner of each photograph. Figure 3a depicts the initial state. The middle state is illustrated in Fig. 3b. Two opposing films bent and came close to each other when the applied pressure was 30 kPa. Finally, the two films came into contact at the top part and formed a tube shape at 40 kPa (Fig. 3c). It is possible to firmly close the tube by applying higher pressure. In total, 40 kPa was applied to the PBAs when the contact parts were closed firmly at the top.

### Characterization

#### Characterization of PBA outputs depending on applied pressure

**Figure 4:**
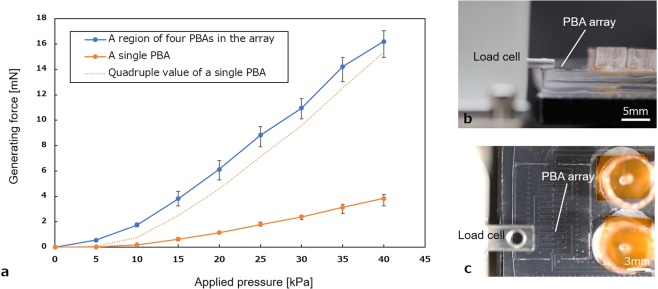
Characterization of generating force depending on supplied pressure (**a)** The generating force of a single bending PBA was measured by a load cell. The standard error is used in (**a**). The generating force increased in accordance with the applied pressure. When measuring the force for the transformation to tube structure, the detecting part of the load cell contacted a region of four PBAs in the array. The value that quadrupled the force by a single PBA is also shown on the dotted line. **(b)** Side view of the measurement setup. **(c)** Top view of the measurement setup. The load cell contacted a region of four PBAs in the PBA array.

The evaluation of the generating force of PBAs is presented in Fig. 4. The standard error is used in Fig. 4a. Figure 4b,c present side and top views of the measurement setup, respectively. First, the generating force of a single bending PBA was measured by a load cell. In such cases, the generating force increased in accordance with the applied pressure. Likewise, the bending angle also increased according to the applied pressure. A single PBA generated 3.8 mN from an applied pressure of 40 kPa. A morphologically transformable structure consisted of an array of PBAs. When measuring the force for the transformation to a tube structure, the detecting part of the load cell contacted a region of four PBAs in the array (Fig. 4c). For the sake of comparison, the value that quadrupled the force by a single PBA is also plotted by an orange dotted line in Fig. 4a. The measured force by the tube structure exhibits a higher value than the quadruple value of the force by a single PBA. This difference suggests that the detected part having four PBAs was also affected by the force from adjacent PBAs in the array.

#### Independent motion control of PBAs for peristaltic motion

**Figure 5:**
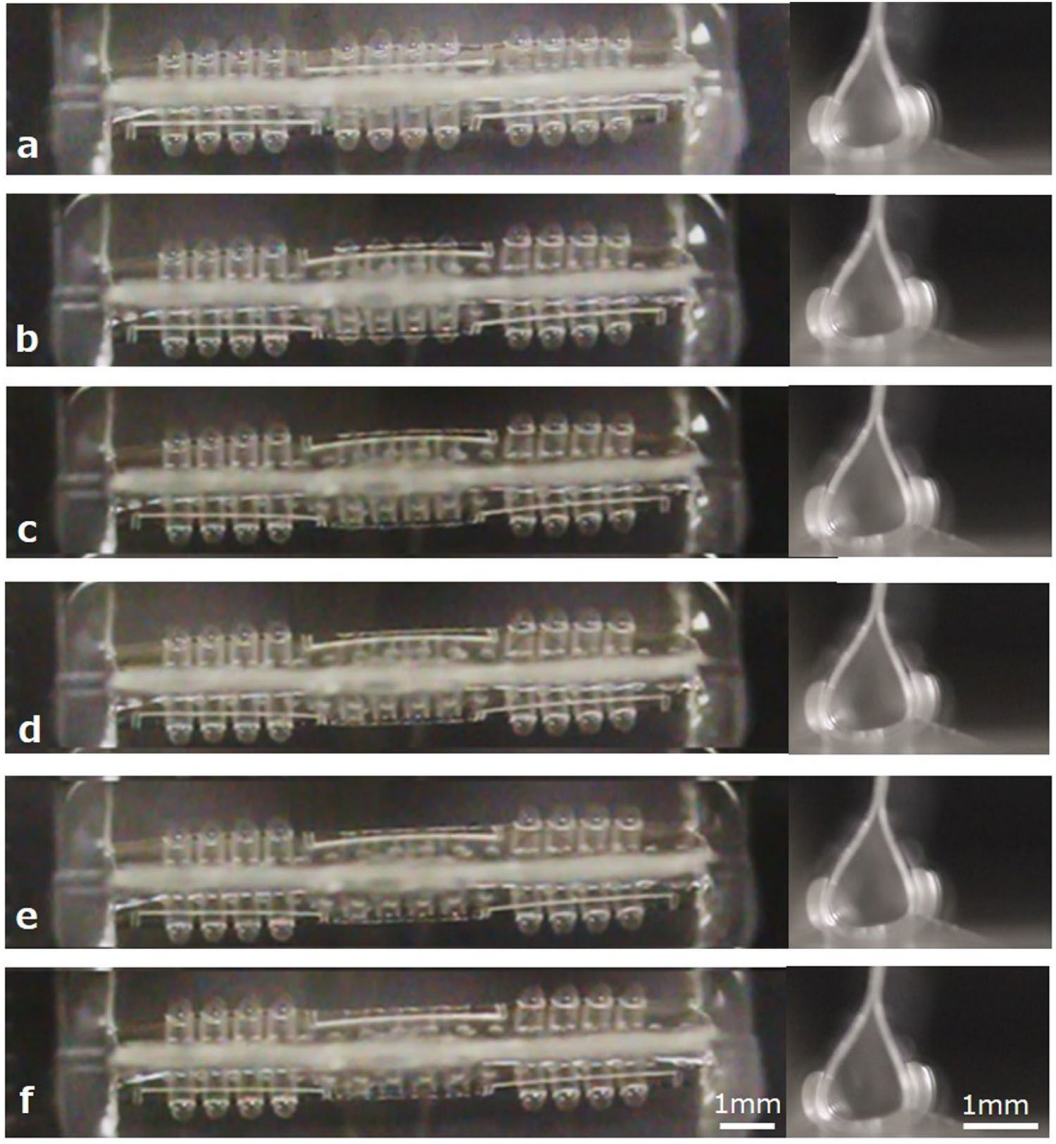
Independent motion control of PBAs toward the peristaltic motion. A whole device was composed of twelve arrays of PBA. The device was divided into three independent sections having four PBAs for each. The independent motion of PBAs in the central section was observed in the experiment. Both top and side views are presented in each photograph. (**a**) Three sections are equally actuated by pressurization to form a tube structure. (**b–e**) The applied pressure to the central section was decreased. PBAs at sections of both ends were kept. (**f**) The applied pressure to the central part was stopped whereas PBAs at both ends were kept.

**Figure 6:**
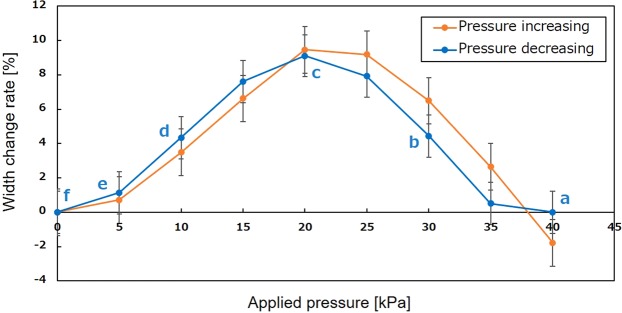
Width change rate at the central section with respect to the applied pressure. The maximum width at the central section was measured when the applied pressure to the central section was decreased. The data (**a–f**) in correspond to Fig. 5a–f, respectively.

The whole device in Fig. 5 is composed of twelve arrays of PBAs. This device was divided into three independent sections with four PBAs each. Performing independent pressure control on each section allows us to explore the possibility of generating higher-order motion, such as peristaltic motion by the small intestine. The independent motion of PBAs in the central section was observed in the experiment as illustrated in Fig. 1c. Figure 5a–f present a series of photographs when the applied pressure to the central section was decreased. Top and side views of the device were presented in Fig. 5. Three sections were equally actuated by the same pressure (Fig. 5a). Then, the applied pressure to the central section was decreased, and the PBAs in the sections at both ends remained unaltered (Fig. 5b–e). Next, the pressure applied to the central part was stopped, and the PBAs in the sections at both ends remained unaltered (Fig. 5f). Figure 6 shows the relationship between the applied pressure and the maximum width of the tube structure. In total, 40 kPa was applied to all three sections to form the tube structure in the initial state (Fig. 5a). The applied pressure to the PBAs at the central section was decreased to 5 kPa from 40 kPa, whereas the applied pressure to the sections at both ends was kept at 40 kPa (Fig. 5e). Finally, the applied pressure to the central section was decreased to 0 kPa (Fig. 5f). A detailed analysis of how the cross-section of the tube structure changed will be discussed in the next section.

## Discussion

### Fundamental characteristics

Connected PBAs affect each other. Therefore, it is effective to design PBAs with high density to obtain high output. In this study, it was possible to increase the force density by increasing the number of PBAs at the same width of the film for the tube structure. However, the performance of each PBA also depends on its own geometry. The rectangular shape of PBA is designed to generate anisotropic deformation along the longitudinal axis. The design of PBA is based on past experimental data for optimization of the performance of PBA. It is also important to consider the surface condition of the internal wall in terms of cell cultures. Consequently, the current design of a rectangular shape employs its long side, which is nine times as long as the short side. Thus, it is important to optimize these related parameters by applying a trade-off between them. In particular, this study designed twelve PBAs (400 μm × 1800 μm × 100 μm) arranged with a regular spacing of 300 μm in each film. It is also important to guarantee the response characteristics of the actuator required for the application of a biomimetic tube. The reported frequency of expansion/contraction motion of a small intestine was less than 1 Hz^35^. The resonant frequency of a PBA for the tube structure with a diameter of 1 mm in this study was estimated at more than 10 Hz, which can mimic the motion of the small intestine. Substantial hysteresis was not observed in the evaluation using a single PBA. In addition, PBA uses pressure control which has advantages in waste or delay of time. The volume of the cavity for PBA and supply channels is small enough to satisfy the requirement of response time for peristaltic motion of organs such as the small intestine.

### Cross-section change of the tube depending on applied pressure

The deformation of the central section of the tube was examined as the applied pressure to the central section decreased. PBAs in all three sections were actuated by the same pressure (40 kPa) to form the tube structure in the initial state. The pressure applied to PBAs in the central section was decreased, whereas the PBAs in the sections at both ends were kept under the initial condition. Top views of the tubular structure at each condition have been captured and shown in Fig. 5 together with corresponding side views. The maximum width of the cross-section was evaluated using top views in Fig. 5. The change rate of the maximum width with respect to the applied pressure was evaluated in Fig. 6. The data indicated by a-f in Fig. 6 correspond to Fig. 5a–f in decreasing the applied pressure, respectively. The standard error is used in Fig. 6. The width increased from a-state to c-state when the applied pressure for the central section was decreased. The width decreased from c-state to f-state when the applied pressure decreased to 0 kPa. The width change rate shows the maximum value at the c-state whereas it shows the minimum value at a-state and f-state. The cross-section extension and contraction of the tube can be applied to generate an oscillation that leads to peristaltic motion. The results in Figs 5 and 6 show that the device has the ability to deform individual sections independently. The cross-section change between a-state and c-state is effective for the pumping motion in terms of pressure controllability. The behavior of the deformation of the device depends on its dimensions. An appropriate optimization of the design by taking into consideration the trade-off between related parameters will provide effective higher-order motion of coordinated simple microactuators.

**Figure 7:**
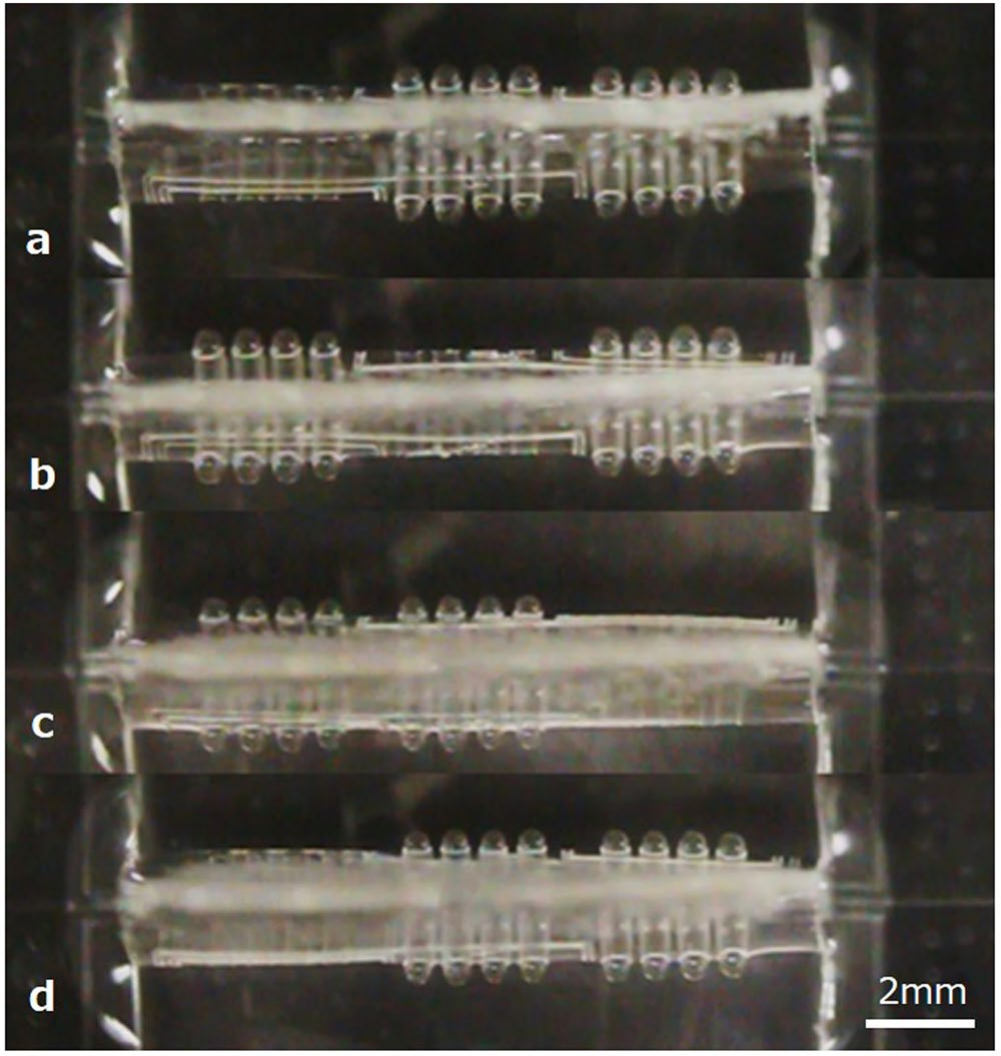
Sequential independent motions of three sections of an array of PBAs toward the peristaltic motion. Individual three sections of the device have four PBAs. Initially, all PBAs of three sections were pressurized to form a tube structure. **(a)** The pressure to PBAs in the left section was decreased whereas other PBAs were kept in the initial state. **(b)** The pressure to PBAs in the central section was decreased whereas PBAs in the left section were pressurized again. **(c)** The pressure to PBAs in the right section was then decreased whereas PBAs in the central section were pressurized again. **(d)** The device returned to the initial state where PBAs in the left section were unpressurized and other PBAs were pressurized.

Figure 7 demonstrates sequential independent motions of three sections of an array of PBAs toward the peristaltic motion. Individual three sections of the device have four PBAs. Initially, all PBAs of three sections were pressurized (40 kPa) to form a tube structure. The pressure to PBAs in the left section was decreased whereas other PBAs were kept in the initial state (Fig. 7a). The pressure to PBAs in the central section was decreased whereas PBAs in the left section were pressurized again (Fig. 7b). The pressure to PBAs in the right section was then decreased whereas PBAs in the central section were pressurized again (Fig. 7c). The device returned to the initial state where PBAs in the left section were unpressurized and other PBAs were pressurized (Fig. 7d). As a result, we can see that coordinated motion of soft PBAs can provide higher-order functions of the tube structure, such as peristaltic motion.

## Methods

A transformable tube structure was fabricated according to our previous work^36^. The structure integrated with PBA was fabricated by photolithography and PDMS replica molding. The main material for PBA was PDMS (Silpot 184, Dow Corning Inc.). The PDMS flat layer and micropatterned PDMS layer were bonded using an imperfect-cured surface in^36^.

Experimental setups for the evaluation of devices are described. Applied pressure to PBAs was controlled by a syringe pump (PUMP33, Harvard Apparatus). The measurement setup of the generating force is shown in Fig. 4b,c. The force was measured by a load cell (LVS-20GA, Kyowa Electronic Instruments Co., Ltd.). The applied pressure to form the tube structure was estimated in advance and applied to the device in the experiments. It was necessary to apply 40 kPa to the PBAs when the contact parts of tube structure were closed at the top. Magnified pictures of the devices were captured by a digital microscope (VHS 500F, Keyence Co.).

## Data Availability

All data generated or analyzed during this study are included in this published article.

## References

[1] Hirano T, Furuhata T, Gabriel KJ, Fujita H (1992). Design, fabrication, and operation of sub-micron gap comb-drive microactuators. J. MEMS.

[2] Sampsell JB (1994). Digital micromirror device and its application to projection displays. Journal of Vacuum Science & Technology B: Microelectronics and Nanometer Structures Processing, Measurement, and Phenomena.

[3] Ataka M, Takeshima N, Omodaka A, Fujita H (1993). Polyimide bimorph actuators for a ciliary motion system. J. MEMS.

[4] Schroth A, Lee C, Matsumoto S, Maeda R (1999). Application of sol-gel deposited PZT film for actuation of 1D and 2D scanners. Sensors and Actuators A.

[5] Kuribayashi, K. & Reversible S. M. A. actuator for micron sized robot. *Proc. IEEE MEMS’90*, 217–221, (1990).

[6] Konishi S, Kawai F, Cusin P (2001). Thin flexible end-effector using pneumatic balloon actuator. Sensors and. Actuators A.

[7] Lu, Y. & Kim, C. J. Micro-finger articulation by pneumatic parylene balloons. *Proc. Transducers’03*, 276–279 (2003).

[8] Jeong OC, Konishi S (2006). All PDMS pneumatic microfinger with bidirectional motion and its application. J. MEMS.

[9] Böhringer, K. F. & Choset, H. *Distributed manipulation*. (Springer, 2000).

[10] Ebefors T, Mattsson JU, Kalvesten E, Stemme G (2000). A robust micro conveyer realized by arrayed polyimide joint actuators. J. MEMS.

[11] Ataka M, Legrand B, Buchaillot L, Collard D, Fujita H (2009). Design, fabrication, and operation of two-dimensional conveyance system with ciliary actuator arrays. IEEE/ASME Trans. on Mechatronics.

[12] Zengerle R, Ulrich J, Kluge S, Richter M, Richter A (1995). A bidirectional silicon micropump. Sensors and. Actuators A.

[13] Stemme E, Stemme G (1993). Valveless diffuser/nozzle-based fluid pump. Sensors and. Actuators A.

[14] Smits JG (1990). Piezoelectric micropump with three valves working periodically. Sensors and. Actuators A.

[15] Jeong OC, Konishi S (2008). Fabrication of a Peristaltic Micro Pump with Novel Cascaded Actuators. J. MEMS.

[16] Jeong OC, Konishi S (2010). Fabrication of Peristaltic Micropump Driven by a Single-Phase Pneumatic Force. Japanese Journal of Applied Physics.

[17] Tripathi D, Jhorar R, Anwar Beg O, Kadir A (2017). Electro-magneto-hydrodynamic peristaltic pumping of couple stress biofluids through a complex wavy micro-channel. J. Molecular Liquids.

[18] Shutko AV, Gorbunov VS, Guria KG, Agladze KI (2017). Biocontractile microfluidic channels for peristaltic pumping. Biomed Microdevices.

[19] Ma T, Sun S, Li B, Chu J (2019). Piezoelectric peristaltic micropump integrated on a microfluidic chip. Sensors and. Actuators A.

[20] Shepherd, R. F. *et al*. Multigait soft robot, *PNAS*, 10.1073/pnas.1116564108, (2011).

[21] Gorissen, B. *et al*. Elastic inflatable actuators for soft robotic applications. *Advanced Materials*, 1604977 (2017). 10.1002/adma.201604977 28949425

[22] Ross M, Pister K (1995). Mcro-widmill for optical scanning and flow measurement. Sensors and Actuators A.

[23] Kaajakari V, Lal A (2003). Thermokinetic actuation for batch assembly of microscale hinged structures. J. MEMS.

[24] Iwase E, Shimoyama I (2005). Multistep sequential batch assembly of three-dimensional ferromagnetic microstructures with elastic hinges. J. MEMS.

[25] Efimovskaya A, Lin YW, Shkel AM (2017). Origami-like 3-D folded MEMS approach for miniature inertial measurement unit. J. MEMS.

[26] Azam A, Laflin KE, Jamal M, Fernandes R, Gracias DH (2011). Self-folding micropatterned polymeric containers. Biomed Microdevices.

[27] Bolanos Quinones VA, Zhu H, Solovev AA, Mei Y, Gracias DH (2018). Origami biosystems: 3D assembly methods for biomedical applications. Adv. Biosys.

[28] Akiyama T, Shono K (1993). Controlled stepwise motion in polysilicon microstructures. J. MEMS.

[29] Akiyama T, Collard D, Fujita H (1997). Scratch drive actuator with mechanical links for self-assembly of three-dimensional MEMS. J. MEMS.

[30] Fan, L., Wu, M. C., Choquette, K. & Crawford, M. Self-assembled microactuated XYZ stages for optical scanning and alignment, *Proc. Transducer’97*, 319–322 (1997).

[31] Fukuta, Y., Collard, D., Akiyama, T., Yang, E. H. & Fujita, H. Microactuated self-assembling of 3D polysilicon structures with reshaping technology, *Proc. IEEE MEMS’97*, 447–481 (1997).

[32] Stolpe AVD, Toonder JD (2013). Workshop meeting report Organs-on-Chips: human disease models. Lab on a chip.

[33] Hua D, Torisawa Y, Hamilton G, Kim H, Ingber DE (2012). Microengineered physiological biomimicry: Organs-on-Chips. Lab on a chip.

[34] Kabirian F, Amoabediny G, Haghighipour N, Salehi-Nik N, Zandieh-Doulabi B (2014). Nitric oxide secretion by endothelial cells in response to fluid shear stress, aspirin, and temperature. Society for biomaterials.

[35] Kim H, Hua D, Hamilton G, Ingber DE (2012). Human gut-on-a-chip inhabited by microbial flora that experiences intestinal peristalsis-like motions and flow. Lab on a chip.

[36] Konishi S, Fujita T (2015). An openable artificial intestinal tract system for the *in vitro* evaluation of medicines. Microsystems & Nanoengineering.

